# Gleeble-Simulated and Semi-Industrial Studies on the Microstructure Evolution of Fe-Co-Cr-Mo-W-V-C Alloy during Hot Deformation

**DOI:** 10.3390/ma11122577

**Published:** 2018-12-18

**Authors:** Yiwa Luo, Hanjie Guo, Jing Guo, Wensheng Yang

**Affiliations:** 1 School of Metallurgical and Ecological Engineering, University of Science and Technology Beijing, Beijing 100083, China; lyw918@126.com (Y.L.); guojing@ustb.edu.cn (J.G.); yws3179608@163.com (W.Y.); 2 Beijing Key Laboratory of Special Melting and Preparation of High-end Metal Materials, Beijing 100083, China

**Keywords:** Fe-Co-Cr-Mo-W-V-C alloy, hot forming, constitutive equation, microstructure evolution, first principles, carbide

## Abstract

Fe-Co-Cr-Mo-W-V-C alloy is one of the most important materials for manufacturing drills, dies, and other cutting tools owing to its excellent hardness. However, it is prone to cracking due to its poor hot ductility during continuous hot working processes. In this investigation, the microstructure characteristics and carbide transformations of the alloy in as-cast and wrought states are studied, respectively. Microstructural observation and first-principles calculation were conducted on the research of types and mechanical properties of carbides. The results reveal that carbides in as-cast Fe-Co-Cr-Mo-W-V-C alloy are mainly Mo_2_C, VC, and Cr-rich carbides (Cr_7_C_3_ and Cr_23_C_6_). The carbides in wrought Fe-Co-Cr-Mo-W-V-C alloy consist of Fe_2_Mo_4_C, VC, Cr_7_C_3_, and a small amount of retained Mo_2_C. For these carbides, Cr_7_C_3_ presents the maximum bulk modulus and B/G values of 316.6 GPa and 2.48, indicating Cr_7_C_3_ has the strongest ability to resist the external force and crack initiation. VC presents the maximum shear modulus and Yong’s modulus values of 187.3 GPa and 465.3 GPa, which means VC can be considered as a potential hard material. Hot isothermal compression tests were performed using a Gleeble-3500 device to simulate the flow behavior of the alloy during hot deformation. As-cast specimens were uniaxially compressed to a 70% height reduction over the temperature range of 1323–1423 K and strain rates of 0.05–1 s^−1^. A constitutive equation was established to characterize the relationship of peak true stress, strain rate, and deformation temperature of the alloy. The calculated results were in a good agreement with the experimental data. In order to study the texture evolution, the microstructures of the deformed specimens were observed, and an optimal deformation temperature was selected. Using the laboratorial optimal temperature (1373 K) in forging of an industrial billet resulted in uniform grains, with the largest size of 17 µm, surrounded by homogenous spherical carbides.

## 1. Introduction

With excellent mechanical properties at high temperatures, Fe-Co-Cr-Mo-W-V-C alloy has been used to replace traditional industrial cutting tool materials. Owing to the addition of molybdenum, chromium, tungsten, and vanadium to around 15 wt.%, a large amount of carbides precipitate in the microstructure during solidification and cooling, which give the cutting tools higher hardness, better wear resistance, and strength [1,2,3]. Although the cobalt (about 8 wt.%) in the alloy is not a carbide-forming element, it contributes to increasing dislocation density, retarding dislocation recovery, and improving high-temperature properties [4,5]. However, the abundant carbides in this high-alloy steel cause a challenge in preventing the generation of the large size of primary carbides. The typical microstructure in the as-cast alloy consists of dendrites surrounded by coarse M_2_C-type eutectic carbides with a networked structure. These coarse primary carbides undergo decomposition, dissolution, transformation, and precipitation during hot deformation, and transform into a more stable form that can be expressed as M_2_C + matrix→M_6_C + MC [6,7,8,9]. Nogueira et al. [10] reported that undecomposed reticular carbides could provide routes for crack propagation and make cracks deeper. Zhou et al. [11] reported that metastable M_2_C carbides could easily be decomposed into large sizes and uneven distributed carbides during hot deformation, which eventually deteriorates the ductility and impact toughness of the alloy. Accordingly, controlling the decomposition of metastable carbides, as well as the dimension and distribution of retained carbides during hot deformations, were found to have a decisive influence on the mechanical properties of the final product.

As is well-known, industrial cutting tool materials are generally produced by the classical route of ingot casting followed by hot forging, rolling, and subsequent heat treatments, i.e., annealing, quenching, and tempering [12], which implies that dynamic deformation of forging and rolling is the focus of whether the reticular carbides are cracked and decomposed adequately or not. Gleeble-simulated research, which is a popular method for the laboratory investigation on the mechanical and thermal behaviors of materials, could imitate hot deformation processes and present the correlation among the flow stress, strain, strain rate, and temperature. In the past decades, many pieces of research have been reported about the deformation behaviors of different materials, i.e., steels [13,14,15], titanium alloys [16,17], magnetic alloys [18], aluminum alloys [19,20], and Ni-base alloys [21,22]. However, studies on the microstructure evolution and the characteristics of carbides of high-alloy tool steels during hot deformation are insufficient as tool steels are sensitive to processing temperatures, so each kind of tool steel has its specific processing parameter related to hot workability.

A tool steel (Fe-Co-Cr-Mo-W-V-C alloy) with wide application prospects was chosen in the present work. Hot isothermal compression tests have been performed on the alloy during upsetting processes in a range of temperatures (1323 K, 1373 K, and 1423 K) and strain rates (0.05 s^−1^, 0.1 s^−1^, 1 s^−1^). Based on the experimental strain-stress data obtained from the compression tests, a constitutive equation was established to characterize the relationship of true stress, strain rate, and temperature during the hot deformation. An optimal processing temperature of Fe-Co-Cr-Mo-W-V-C alloy was selected for hot working. In addition, to study the texture evolution and carbide transformation, the microstructures of the as-cast ingot, deformed specimens, and the wrought alloy were observed by light microscope (LM) and scanning electron microscope (SEM).

## 2. Materials and Methods

### 2.1. Material

Materials used in this investigation were provided by the pilot plant of Central Iron & Steel Research Institute (Beijing, China). The as-cast alloy was produced in an electroslag remelting (ESR) furnace (Beijing Cisri-Gaona Materials & Technology Co., Ltd., Beijing, China), and its size was 160 mm in diameter and 1000 mm in length. The chemical composition of the alloy was given as follows: C 1.02, Mn 0.28, Si 0.35, Co 8.05, Cr 4.31, Mo 8.95, V 1.32, W 1.15, Fe Bal (in wt.%). The wrought alloy, a billet with a section size of 80 × 80 mm, was obtained by forging of the ESR ingot. The ESR ingot was preheated in stages to 893 and 1123 K and isolated for 1.5 h at each temperature, then homogenized at 1373 K for 2 h and forged into a billet in 3 passes.

### 2.2. Compression Simulation Test

Uniaxial compression tests were carried out on the Gleele-3500 thermal simulator to evaluate the hot deformation behavior. Cylindrical specimens with a diameter of 8 mm and height of 12 mm were machined from the as-cast ESR ingot, and isothermally compressed at the strain rates of 0.05, 0.1, and 1 s^−1^ and in the temperature range of 1323, 1373, and 1423 K until the engineering compressive strain reached 70%. The heating and cooling rates were fast enough to avoid precipitation while achieving the testing temperature. To avoid inhomogeneous deformation, tantalum foils and graphite foils were inserted between the cylindrical specimen and the compression tool. After the experiment, flow curves of the Fe-Co-Cr-Mo-W-V-C alloy were measured, and the longitudinal microstructures of the deformed specimens were obtained from the vertical section of the semi-cylindrical samples.

### 2.3. Microstructure Characterization

To study the microstructure evolution, the as-cast ingot, wrought alloy, and specimens after the hot isothermal compression test were metallographically ground, polished, and observed by LM and SEM with the function of the energy dispersive spectroscopy (EDS) (Carl-Zeiss, Oberkochen, Germany) in backscattering mode. The grain size of the specimens were measured by Image-Pro Plus software (Image-Pro Plus 6.0, Media Cybernetics, Rockville, MD, USA). Specimens for grain size measurement were polished and etched using a Nital etchant (Ethanol + 4 vol.% Nitric acid) to reveal the grain boundary, and then measured according to GB/T 6394-2002. The linear truncation method was performed to determine the grain size by measuring the number of cutoff points between a certain length of line and austenite grain boundaries. A transmission electron microscope (TEM) (FEI, Hillsboro, AL, USA) was employed to identify the secondary carbides with small sizes. The flaky wrought specimen with dimensions of 10 × 10 mm was ground to a thickness of 50 μm and ion-bombarded to a thin foil, suitable for TEM-operating.

### 2.4. Calculation of Mechanical Properties of Carbides

First-principle calculation was performed based on the pseudopotential plane-wave within the framework of density functional theory using the Vienna Ab-initio Simulation Package (VASP). In the calculations of VC, Cr_7_C_3_, Cr_23_C_6_, Fe_2_Mo_4_C, and Mo_2_C, the cutoff energies were all set at 350 eV and their numbers of k-point were 6 × 6 × 6, 6 × 4 × 2, 5 × 5 × 5, 10 × 10 × 10, and 5 × 4 × 5, respectively. After getting equilibrium geometry configures, the “stress-strain” method was applied to obtain the elastic constants. Based on Hooke’s law and Voigt-Reuss mode, the mechanical properties of each kind of carbide in Fe-Co-Cr-Mo-W-V-C alloy was calculated.

## 3. Results

### 3.1. As-Cast and Wrought Microstructures

**Figure 1:**
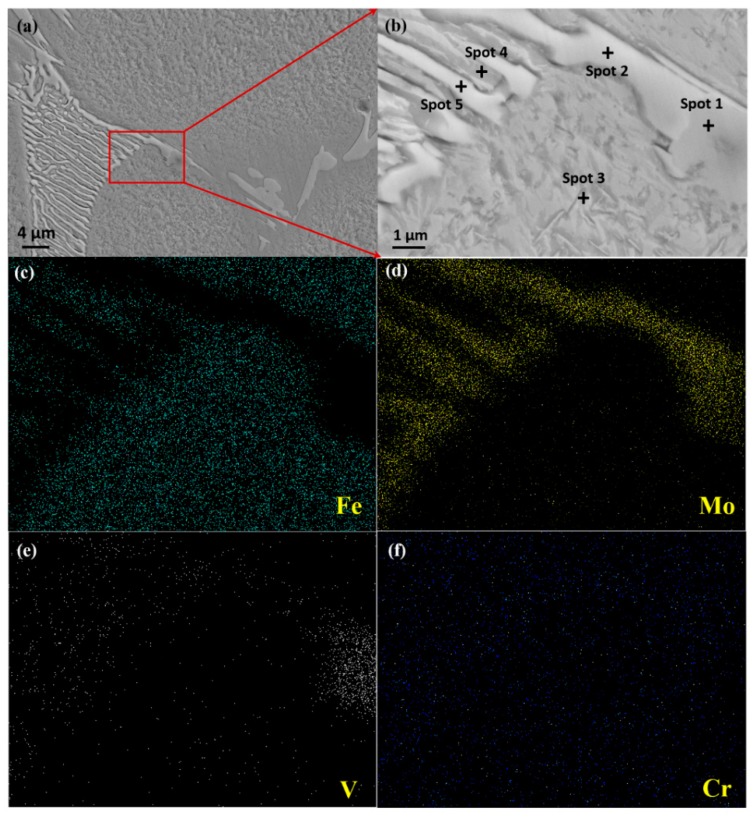
(**a**) Scanning electron microscope (SEM) micrograph of as-cast Fe-Co-Cr-Mo-W-V-C alloy, (**b**) detail of the microstructure in the red frame of (**a**), (**c**) distribution map of Fe, (**d**) distribution map of Mo, (**e**) distribution map of V, (**f**) distribution map of Cr.

Multiple transition metal elements, such as W, Mo, Cr, and V, have the potential to form a variety of carbides, i.e., MC, M_2_C, M_6_C, M_7_C_3_, and M_23_C_6_, during the solidification and cooling of the alloy [23,24,25,26]. A backscattered-electron image of the microstructure of the as-cast Fe-Co-Cr-Mo-W-V-C alloy is shown in Figure 1a, with a higher magnification image of eutectic carbides in Figure 1b. Figure 1c–f illustrate EDS chemical distribution maps of Fe, Mo, V, and Cr. In the as-cast condition (Figure 1a), the high alloy content in the alloy inherently gives rise to carbide segregation at grain boundaries and a skeleton microstructure of primary carbides. According to the previous research of the authors [27], the microstructure is composed of α-ferrite, austenite, lamellar eutectic carbides, and blocky carbides in the matrix. Table 1 lists the chemical compositions of the marked matrix and eutectic carbides of different shapes analyzed by EDS. Spot 1 was taken on blocky carbide, and spots 2 and 5 were taken on lamellar carbides with different widths. Spots 3 and 4 were taken in the matrix, where “3” is further away from carbides than “4”. The matrix far away from carbides exhibits a larger amount of Fe, but fewer amount of Mo than that next to the carbides. Besides, the proportion of Co in the matrix is almost the initial cobalt content of the alloy, which confirms that Co is not a carbide-forming element, but is used to improve the mechanical properties of the matrix. According to Table 1 and Figure 1c–f, it could be considered that lamellar eutectic carbides are Mo-rich M_2_C with a small quantity of Fe, W, Cr, and V. Rod-like carbides are MC that mainly consist of V and Mo. This is in agreement with the literature [28,29,30] for as-cast microstructures of tool steels. The distribution of Cr (Figure 1f) presents a wider area compared with the eutectic carbide network and is concentrated in a certain region, which indicates the existence of fine Cr-rich carbides. Based on the authors’ previous study [31], the types of Cr-rich carbides in alloy are generally M_7_C_3_ and M_23_C_6_.

**Figure 2:**
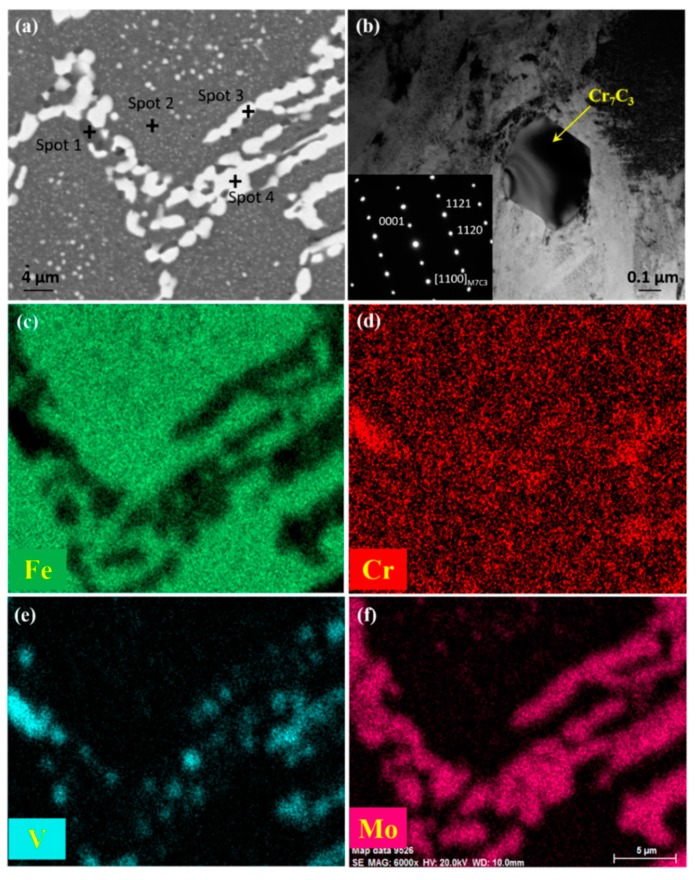
(**a**) SEM micrograph of wrought Fe-Co-Cr-Mo-W-V-C alloy, (**b**) transmission electron microscope (TEM) image of a typical secondary carbide with its selected area electron diffraction (SAED) pattern, (**c**) distribution map of Fe, (**d**) distribution map of Cr, (**e**) distribution map of V, (**f**) distribution map of Mo.

A backscattered-electron image of the microstructure of the wrought Fe-Co-Cr-Mo-W-V-C alloy is shown in Figure 2a. Continuous bright spherical carbides were widely distributed at the grain boundaries, with black carbides embedded in them. Meanwhile, a large number of small carbides were present in the matrix. TEM was employed to identify the type of these carbides because it is difficult to distinguish precipitates less than 1 μm using SEM and EDS. The TEM image of a carbide which was frequently observed and its selected area electron diffraction (SAED) pattern is shown in Figure 2b. Figure 2c–f present the chemical distribution maps of Fe, Cr, V, and Mo. Table 2 illustrates the chemical compositions of the marked matrix and typical eutectic carbides analyzed by EDS. Spots 1 and 2 were taken on the dark carbide and matrix, respectively. Spots 3 and 4 were taken on bright carbides, where “4” is more inland to the carbides than “3”. As shown in Figure 2a, the carbide network structure typical for the as-cast microstructure was crushed. The carbide lamellas were characterized by constriction, fracture, and spheroidization. The sizes of the bright sphere carbides were approximately 3–5 µm. Dark carbides were much smaller than bright carbides, with sizes below 3 µm. From EDS map scanning of the wrought microstructure, different distributions of V (Figure 2e) and Mo (Figure 2f) revealed two carbide types, Mo-rich carbides and V-rich carbides. More uniform distribution of Cr (Figure 2d) throughout the matrix points out the existence of Cr-rich secondary carbides, which is confirmed by TEM observation, as shown in Figure 2b. Cr-rich carbides are hexagonal M_7_C_3_ (Cr_7_C_3_) with the lattice constant of a = b = 6.9576 Å, c = 4.4511 Å. According to Table 2, the dark carbide rich in V is most likely of MC (VC) type. The carbide at Spot 3 which mainly consisted of Mo and Fe was M_6_C (Fe_2_Mo_4_C), while the carbide at Spot 4 with an increased content of Mo was M_2_C (Mo_2_C). As previously reported by other researchers [32,33,34], metastable M_2_C carbide is decomposed and converted during the heating and deformation process as follows: M_2_C + matrix → M_6_C + MC. The transformation starts on the boundary between the carbides and the matrix, and then grows towards the inner region. When the reaction is incomplete, the M_2_C that has not been transformed is retained in the microstructure. Table 2 shows that M_2_C carbide is richer in V than M_6_C carbide, which means that the content of vanadium controls the amount of MC carbides. During the reaction, vanadium dissolves out of M_2_C carbides to make room for the formation of M_6_C carbides. Hence, the solubility of vanadium decreases, and precipitates as V-rich MC carbides next to M_6_C. This provides a reasonable explanation for why MC carbides mostly precipitates between M_6_C carbides.

### 3.2. Mechanical Properties of the Carbides

The hot workability and toughness of Fe-Co-Cr-Mo-W-V-C alloy are greatly influenced by the carbides, whose mechanical properties are closely related to the elastic constants. Hence, a stress-strain approach is employed to calculate elastic constants based on the first-principles calculation in this study. The main advantage of this method is that the required strain patterns can be greatly reduced for the low-symmetry crystal structures which usually have large number of independent elastic constants [35]. The calculated cell parameters and independent elastic constants (*C*_ij_) are illustrated in Table 3. According to Hooke’s law and the Voigt-Reuss-Hill model [36,37], the bulk modulus (*B*), shear modulus (*G*), and Young’s modulus (*E*) for polycrystalline crystal can be estimated as follows:

Hexagonal system (Mo_2_C, Cr_7_C_3_):

(1) $$B=[2(C_{11}+C_{12})+4C_{13}+C_{33}]/9$$

(2) $$G=(C_{11}+C_{12}+2C_{33}-4C_{13}+12C_{44}+12C_{66})/30$$

(3) $$E=\frac{9BG}{\left(3B+G\right)}$$

Cubic system (Fe_2_Mo_4_C, VC, Cr_23_C_6_):

(4) $$B=(C_{11}+2C_{12})/3$$

(5) $$G=(C_{11}-C_{12}+3C_{44})/5$$

(6) $$E=\frac{9BG}{\left(3B+G\right)}$$

The calculated results of elastic parameters that depict the elastic properties are given in Table 4. The bulk modulus, which reflects the compressibility of the solid under hydrostatic pressure, are 281.84, 229.74, 300.74, 316.61, and 147.71 GPa for Mo_2_C, Fe_2_Mo_4_C, VC Cr_7_C_3_, and Cr_23_C_6_, respectively. Cr_7_C_3_ presents the largest value in these five carbides due to its strong covalence of Cr–C, implying that the capacity of Cr_7_C_3_ to resist the external force is the best. The values of the shear modulus of all calculated carbides form the following sequence: VC > Mo_2_C > Cr_23_C_6_ > Cr_7_C_3_ > Fe_2_Mo_4_C. The ratio of B/G is frequently used to evaluate the ductility of the precipitates. It is supposed that B/G is less than 1.75 for brittle precipitates and greater than 1.75 for the metallic compound [38]. In the present work, the calculated results reveal that Fe_2_Mo_4_C, Mo_2_C, Cr_7_C_3_ and Cr_23_C_6_ are all conducive to the ductility of the alloy except for VC. In addition, Cr_7_C_3_ is more ductile than other carbides, indicating its tough nature. The maximum value of Young’s modulus is 465.3 for VC, who has a remarkable contribution to the hardness of the alloy. VC is considered as a potential hard material; however, its obvious brittleness can also deteriorate the mechanical properties of the alloy.

### 3.3. Stress-Strain Curves and Microstructure Evolution

#### 3.3.1. Stress-Strain Curves

**Figure 3:**
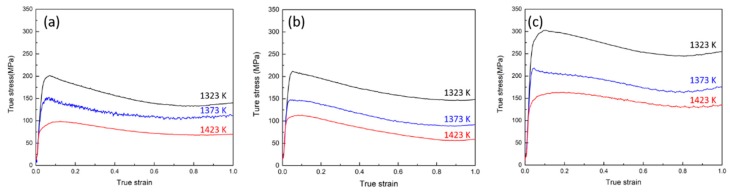
True stress-strain curves of Fe-Co-Cr-Mo-W-V-C alloy during hot deformation at strain rates of (**a**) 0.05, (**b**) 0.1, and (**c**) 1 s^−1^.

Figure 3 illustrates a set of experimental flow curves about the effect of temperature and strain rate on the true stress of the as-cast microstructure. Flow curves were obtained in the temperature range between 1323 and 1423 K at strain rates from 0.05 to 1 s^−1^. All flow stress increases quickly and then declines to some degree after reaching the maximum. The competing deformation mechanisms indicated by the flow curves are work hardening, dynamic recovery, and dynamic recrystallization mechanisms [44]. At the initial state, the accumulation of rapid dislocations results in the growth of flow stress. As the deformation progresses, dynamic recrystallization occurs when the accumulated dislocation density exceeds a critical strain, which decreases the work hardening rate. The flow stress exhibits a continuous reduction with the increasing strain due to the decreased effective stress and increased softening. When the work hardening and dynamic softening are at the same level, the flow curves keep a stable state and account this moment as the final state [45,46,47].

As shown in Figure 3, the flow stress is sensitive to the temperature and strain rate. The peak flow stress value increases with increasing strain rate or decreasing deformation temperature. At a given strain rate, dynamic softening is promoted due to the increase in temperature, which results in a decrease in flow stress. At the same temperature, the dislocation accumulation rate accelerates with the increase in strain rate, which leads to an increase in flow stress.

One of the most familiar equations that could reveal the relationship of flow stress, temperature, and strain rate during hot deformation at a given strain is expressed as follows [48]:

(7) $$\dot{\varepsilon }=A{\left[\sinh(\alpha \sigma )\right]}^{n}\exp(-\frac{Q}{RT})$$

where $\dot{\varepsilon }$ is the strain rate (s^−1^), *A* is the material constant, α is the inverse stress that indicates that the potential relation between stress and strain switches to an exponential one (MPa^−1^), *σ* is the peak true stress (MPa), *n* is the creep exponent, *Q* is the apparent activation energy for hot deformation (KJ/mol), *R* is the gas constant (8.314 J·mol^−1^·K^−1^), and *T* is the temperature (K).

Equation (7) can be simplified as Equation (8) at low stress level and Equation (9) at high stress level [49,50]:

(8) $$\dot{\varepsilon }=A_{1}\sigma^{n_{1}}\exp(-\frac{Q}{RT})$$

(9) $$\dot{\varepsilon }=A_{2}\exp(\beta \sigma )\exp(-\frac{Q}{RT})$$

where *β* and *n*_1_ are constants that are independent of temperature and strain rate.

Equation (10) can be derived by taking the natural logarithm of Equation (7):

(10) $$\ln\dot{\varepsilon }-\ln A+\frac{Q}{RT}=n\ln[\sinh(\alpha \sigma )]$$

At a given temperature, *n* can be expressed as:

(11) $$n={\left[\frac{\partial \ln\dot{\varepsilon }}{\partial \ln[\sinh(\alpha \sigma )]}\right]}_{T}$$

At a given$\;\dot{\varepsilon }$, *Q* can be expressed as:

(12) $$Q=Rn{\left[\frac{\partial \ln[\sinh(\alpha \sigma )]}{\partial T^{-1}}\right]}_{\dot{\varepsilon }}$$

**Figure 4:**
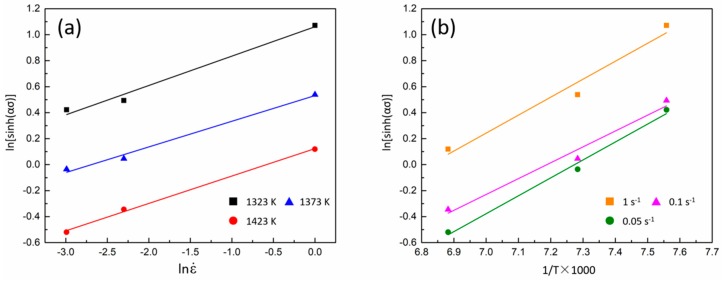
Correlation of ln[sinh(*ασ*)] and (**a**) ln$\dot{\varepsilon }$, and (**b**) 1/*T*.

Hence, *Q* can be calculated from the slope of the linear fitting of ln$\dot{\varepsilon }$ against ln[sinh(*ασ*)] as shown in Figure 4a, and the slope of the linear fitting of ln[sinh(*ασ*)] against 1/*T,* as shown in Figure 4b. From Figure 4a, it can be seen that a linear function is obtained at every temperature, and the slope of each is similar at every set of the test, which indicates that the value of *n* is quite independent of the temperature. Through the calculation of the data in Figure 4b, the average value of the activation energy (*Q*) was found to be 522.042 kJ/mol.

Function *Z* can be defined as:

(13) $$\ln Z=\ln\dot{\varepsilon }+\frac{Q}{RT}$$

Therefore, Equations (7)–(9) can be expressed as follows:

(14) lnZ=lnA+nln[sinh(ασ)]

(15) $$\ln Z=\ln A_{1}+n_{1}\ln\sigma$$

(16) $$\ln Z=\ln A_{2}+\beta \sigma$$

**Figure 5:**
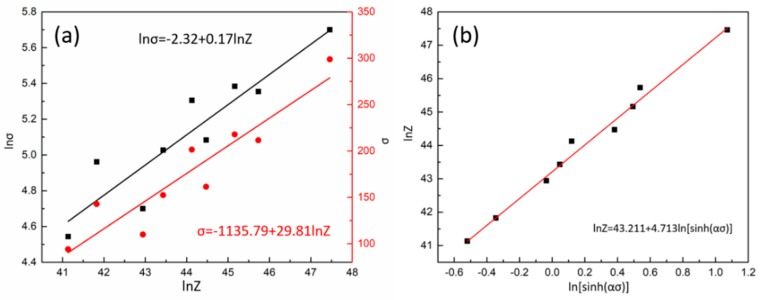
Correlation of ln*Z*, (**a**) ln*σ*, and *σ*, and (**b**) ln[sinh(*ασ*)].

Figure 5a presents the relationship of ln*Z*, *σ,* and ln*σ* procured from experimental data. The slope of the functions obtained from linear fitting of a dataset were precisely the value of 1/*β* and 1/*n*_1_, respectively. 1/*β* was equal to 29.81 and 1/*n*_1_ was equal to 0.17. The value of the coefficient α was determined by *β*/*n*_1_ = 0.006.

Figure 5b presents the relationship of ln*Z* and ln[sinh(*ασ*)]. The slope and intercept of the function obtained from linear fitting of a dataset were precisely the value of n and ln*A*, respectively. *n* was equal to 4.713 and *A* was equal to 5.838 × 10^18^. According to the Reference [51], the value of the creep exponent is close to 5 when deformation is controlled by the glide and climb of dislocations. As a result, the correlation of peak true stress, temperature, and strain rate during hot deformation in Fe-Co-Cr-Mo-W-V-C alloy can be expressed as:

(17) $$\dot{\varepsilon }=5.838\times 10^{18}{\left[\sinh(0.006\sigma )\right]}^{4.713}\exp(-\frac{522.042}{RT})$$

**Figure 6:**
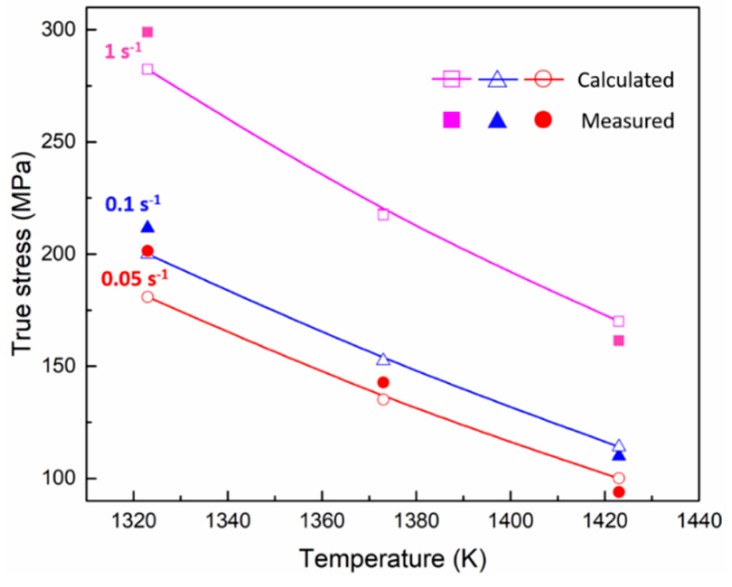
Comparison of true stress between experimental data and calculated results.

Figure 6 illustrates the experimental data from the hot deformation test and the calculated data from Equation (17). The calculated results are in good agreement with the experimental data. This function could be used to select an appropriate temperature and strain rate for hot deformation to improve the hot workability of Fe-Co-Cr-Mo-W-V-C alloy.

#### 3.3.2. Microstructure Evolution

**Figure 7:**
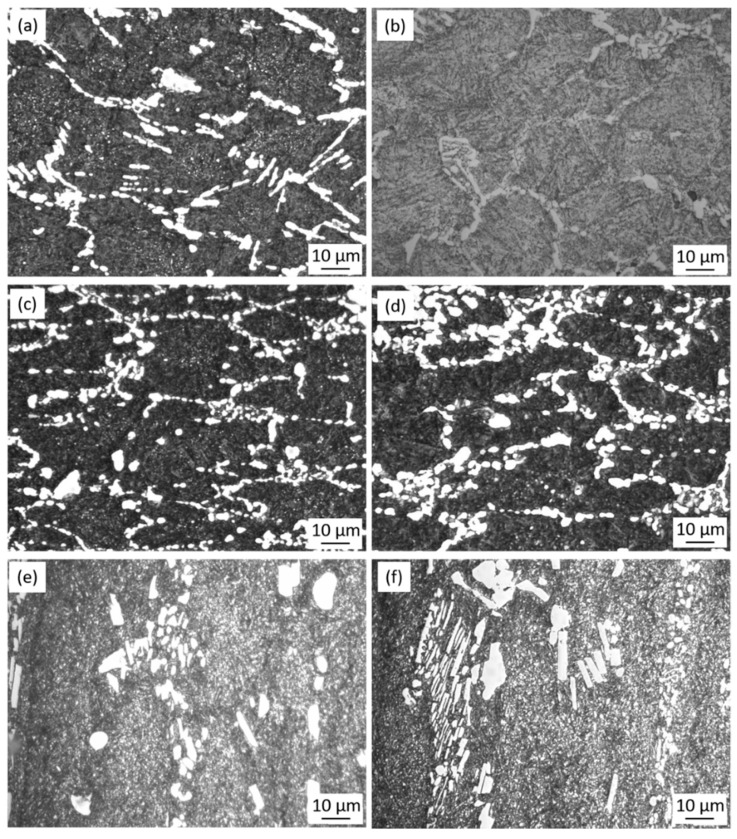
Microstructures of the deformed specimens at 1323 K with a strain rate of (**a**) 0.05 and (**b**) 1 s^−1^, at 1373 K with a strain rate of (**c**) 0.05 and (**d**) 1 s^−1^, and at 1423 K with a strain rate of (**e**) 0.05 and (**f**) 1 s^−1^.

**Figure 8:**
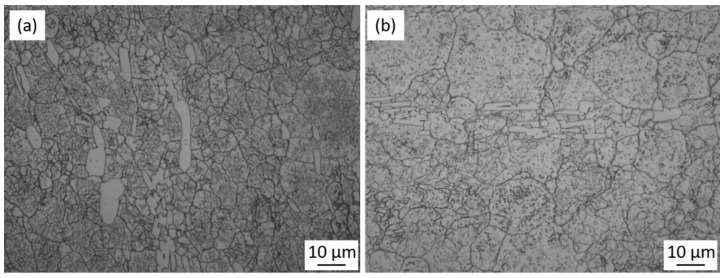
Microstructures of the deformed specimens at a strain rate of 0.1 s^−1^ and a temperature of (**a**) 1373 and (**b**) 1423 K.

A significant contribution towards the increase of stress at the same strain was a mechanical dispersion of carbides in the matrix, which acted as pinning particles during grain growth and strengthened the matrix. Figure 7 presents a series of the micrographs of the as-cast specimens at strain rates of 0.05 and 1 s^−1^ under different temperatures. As shown in Figure 7a,b, the eutectic carbides are not totally crushed under 1323 K and the network morphology of the as-cast microstructure still exists. Besides, at the same temperature, differences in carbide distribution can be seen with the change of strain rate. For example, carbides at the strain rate of 1 s^−1^ (Figure 7d) have variegated sizes and uneven distribution because there is not enough time for the dissolution and homogenization of carbides. On the contrary, more uniform carbide distribution is observed in Figure 7c with a strain rate of 0.05 s^−1^ due to the sufficient time for homogenization. Similar differences in the homogeneity of carbides are found at other experimental temperatures, 1323 and 1423 K. It could be considered that initial deformation of the as-cast microstructures should be conducted with a reduced strain rate. In addition, the amount of the eutectic carbides dissolved into the matrix increased as the temperature increased, resulting in a decrease of the total amount of carbides in the microstructure (Figure 7a,c,e). Figure 8 reveals the micrographs of the as-cast specimens with visible grain boundaries under different heat processing temperatures. It can be seen that a smaller grain size was obtained under a reduced temperature. The hot deformation microstructure at 1373 K with the strain rate of 0.1 s^−1^ exhibits fine and uniform grains, with an average grain size of 11 µm (Figure 8a). As can be seen in Figure 7, as the temperature increases, the carbides are dissolved into the matrix in large quantities, resulting in an insufficient amount of carbides pinning at the grain boundaries. Consequently, the grains in the deformation microstructure performed at the temperature of 1423 K are large and uneven when the strain rate is the same at 0.1 s^−1^, with the average grain size growing up to 18 µm (Figure 8b). It could be considered that a wrought alloy should be conducted at an appropriate temperature that ensures the decomposition of network carbides, and simultaneously avoids excessive growth of grain size. The laboratory hot deformation tests revealed that 1373 K is the optimal temperature at which homogeneous carbides and fine grains can be obtained.

**Figure 9:**
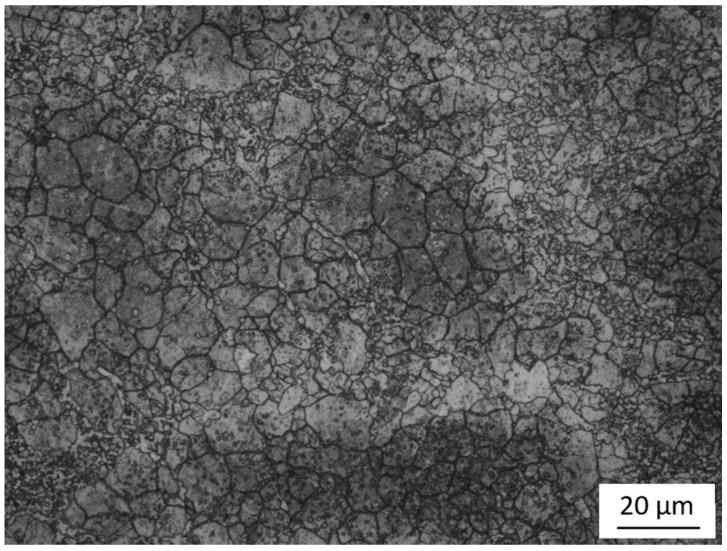
Microstructure of the semi-industrial wrought Fe-Co-Cr-Mo-W-V-C alloy.

A semi-industrial experiment was performed based on the laboratory-determined temperature of hot deformation. An as-cast ESR ingot was forged into a square billet with a diameter of 80 × 80 mm. The microstructure of the wrought billet consisted of fine and uniform grains with a largest size of 17 µm, and surrounded by homogenous spherical carbides, as shown in Figure 9. Besides, the eutectic carbides were commendably crushed at the grain boundaries and the secondary carbides were observed inside the grains.

## 4. Conclusions

Based on the results of the experimental research and theoretical calculations, the following conclusions can be made:

1. The carbides in as-cast Fe-Co-Cr-Mo-W-V-C alloy are principally Mo-rich M_2_C (Mo_2_C), with a certain amount of V-rich MC (VC) and Cr-rich Cr_7_C_3_ and Cr_23_C_6_ carbides. The carbides in wrought Fe-Co-Cr-Mo-W-V-C alloy consist of Mo-rich M_6_C (Fe_2_Mo_4_C), VC, Cr_7_C_3_, and a small amount of retained Mo_2_C.
2. Of the five carbides, Cr_7_C_3_ had the maximum bulk modulus and B/G values of 316.6 GPa and 2.48, indicating that Cr_7_C_3_ has the strongest ability to resist external force and crack initiation. VC had the maximum shear modulus and Yong’s modulus values of 187.3 GPa and 465.3 GPa, which implies VC can be considered as a potential hard material. However, its obvious brittleness can also deteriorate the mechanical properties of the alloy.
3. True stress-strain curves are sensitive to the deformation temperature and strain rate. At a given temperature, the flow stress increases with increasing strain rate. At a given strain rate, the flow stress increases with a decrease in temperature.
4. A constitutive equation to describe the relationship of peak true stress, strain rate, and deformation temperature for Fe-Co-Cr-Mo-W-V-C alloy was established and expressed as:
  (18) $$\dot{\varepsilon }=5.838\times 10^{18}{\left[\sinh(0.006\sigma )\right]}^{4.713}\exp(-\frac{522.042}{RT})$$
  The use of this equation allows one to predict the true stress under different hot working conditions.
5. Under the same strain rate, with an increase of deformation temperature, the network of carbides are broken more completely and better dissolved into the matrix. Because the diffusion rate increases with increasing temperature, which impedes the disruption of grain boundaries in the process of hot deformation and also enhances the grain boundary migration, the average grain size can increase from 11 μm (1373 K) to 18 μm (1423 K).
6. Employing the laboratorial optimal temperature (1373 K) in forging an industrial square billet resulted in uniform grains, with the largest size of 17 µm, surrounded by homogenous spherical carbides.

## Figures and Tables

**Table 1:** Chemical compositions of the marked spots in Figure 1 analyzed by energy dispersive spectroscopy (EDS) (in wt.%).

Spot	C	Fe	Mo	W	Co	Cr	V
1	13.27 ± 1.80	16.70 ± 6.70	24.28 ± 1.16	2.39 ± 0.39	-	6.19 ± 0.19	37.17 ± 7.17
2	12.59 ± 2.34	8.68 ± 0.64	53.41 ± 1.49	7.66 ± 0.66	-	9.38 ± 0.38	8.28 ± 1.28
3	7.22 ± 1.22	74.69 ± 0.17	6.07 ± 0.79	-	7.67 ± 0.16	3.89 ± 0.89	0.46 ± 0.46
4	6.03 ± 0.39	67.53 ± 0.55	11.64 ± 1.64	2.37 ± 0.37	6.85 ± 0.85	4.57 ± 0.57	1.01 ± 0.14
5	15.02 ± 5.02	16.05 ± 6.05	46.66 ± 6.66	8.34 ± 0.34	0.31 ± 0.31	6.16 ± 0.16	7.46 ± 0.66

**Table 2:** Chemical compositions of the marked spots in Figure 2 analyzed by EDS (in wt.%).

Spot	C	Fe	Mo	W	Co	Cr	V
1	13.24 ± 3.24	10.97 ± 0.81	21.87 ± 1.47	2.34 ± 0.43	-	5.78 ± 0.78	45.80 ± 5.90
2	5.47 ± 0.47	75.19 ± 5.19	5.48 ± 0.48	1.87 ± 0.19	7.58 ± 0.58	3.78 ± 0.78	0.63 ± 0.63
3	7.43 ± 1.73	34.09 ± 4.09	39.40 ± 1.40	8.96 ± 0.96	1.66 ± 0.24	5.05 ± 0.53	3.41 ± 0.41
4	11.18 ± 1.18	11.76 ± 1.76	51.73 ± 1.73	8.90 ± 0.90	-	6.93 ± 0.93	9.50 ± 0.15

**Table 3:** Calculated cell parameters in Å and elastic constants C_ij_ of the carbides.

Carbides	Space Group	a	b	c	C_11_	C_12_	C_13_	C_33_	C_44_	C_66_
Mo_2_C	P6_3_/mmc	3.060	3.060	4.655	506.2	138.1	193.3	474.3	184.0	132.1
Fe_2_Mo_4_C	Fd$\bar{3}$m	11.422	11.422	11.422	391.4	148.9	-	-	106.1	-
VC	Fm$\bar{3}$m	4.175	4.175	4.175	611.5	145.3	-	-	156.8	-
Cr_7_C_3_	P6_3_/mmc	6.957	6.957	4.451	504.2	211.7	254.0	402.0	146.2	130.6
Cr_23_C_6_	Fm$\bar{3}$m	10.754	10.7546	10.754	485.2	200.0	-	-	139.6	-

**Table 4:** Bulk modulus *B*, Shear modulus *G*, ratios of *B*/*G*, and Young’s modulus *E* of eutectic carbides (GPa).

Carbides	*B*	*G*	*B/G*	*E*
Mo_2_C	281.8(336.0 ^g^)	153.8(181.6 ^g^)	1.83	390.4(461.6 ^g^)
Fe_2_Mo_4_C	229.7	112.2	2.05	289.4
VC	300.7(293.1 ^g^,290.9 ^b^)	187.3(176.9 ^g^,215.5 ^b^)	1.61	465.3(441.7 ^g^,518.5 ^b^)
Cr_7_C_3_	316.6(300.6 ^a^,311.7 ^e^)	127.5(118 ^a^)	2.48	337.2(313 ^a^)
Cr_23_C_6_	295.1(282.3 ^a^,300 ^c^,294 ^f^)	140.8(145.4 ^a^,136 ^d^,137 ^c^)	2.09	364.5(357 ^c^,372.3 ^a^)

a–g: Refs. [30,38,39,40,41,42,43].
